# Continuity of care: important, but only the first step

**DOI:** 10.1186/2045-4015-1-22

**Published:** 2012-05-23

**Authors:** Janet M Corrigan

**Affiliations:** 1 President and CEO, National Quality Forum, 1030 15th Street, NW, Washington, DC, 20005, USA

**Keywords:** Quality primary care, Care coordination, Continuity of care

## Abstract

Continuity of care is critical to achieving the best outcomes, especially for patients with chronic conditions. Israel’s strong commitment to primary care as a central organizing concept of the health system, accompanied by investments in health information technology and training primary care physicians, has contributed to its impressive levels of continuity of care. Taking the next steps toward a comprehensive system of patient- and population-centered care for proactive management of patients with chronic conditions has much potential to further enhance outcomes and reduce costs.

## 

Continuity of care and care coordination is a critical issue in virtually all health care systems. Worldwide, chronic disease accounts for an estimated 63% of deaths [1]. The toll of managing chronic illness will only increase as populations continue to age. Further, the rapid pace of scientific discovery and technological innovation, accompanied by specialization and greater intensity of health care services, has increased the numbers of clinicians and settings involved in the care process and the complexity of navigating the health care system.

In the United States, the Secretary of Health and Human Services promulgated a National Quality Strategy in 2011 to focus and align improvement efforts around six priorities, one of which is promoting effective communication and care coordination [2]. Inadequate care coordination – incomplete communication or collaboration across people, functions, and sites – contributes to poor quality, unsafe care, and waste [3]. For example, nearly one in five elderly patients is readmitted to the hospital within 30 days, and three-quarters of those readmissions are potentially preventable, costing the U.S. upwards of $12 billion annually [4,5]. Each year, more than 700,000 patients are treated for adverse drug events in U.S. hospital emergency departments and failure to manage and reconcile medications prescribed by multiple physicians is a major contributing factor [6].

The study by Dreiher et al. [7] addresses this important and timely topic and defines continuity of care as “consistent, ‘seamless’ treatment over time involving various healthcare providers and settings”. Continuity of care is operationalized using a variety of indices that measure the propensity to receive services from the same physician. The study found continuity of care in Clalit Health Services to be relatively high compared with levels found in the United States and England. A significant association was found between continuity of care and a decrease in the number and cost of emergency department visits and an increase in the number and cost of medical consultation visits.

A growing body of evidence supports the importance of having a strong primary care base for patients to achieve the best outcomes and to contain costs. Friedberg et al. have identified three dimensions of primary care: [8]

· Specialty of Provider. Training providers as generalists including physicians (e.g., pediatricians, general internists) and non-physicians (primary care nurse practitioners).

· Functions. Ensuring that certain essential functions are performed by the primary care provider, such as serving as a point of first contact for new health problems and assuming responsibility for coordination of care across providers and settings and over time.

· Orientation of Systems. Building a care environment that supports primary care; for example maintaining an appropriate balance between primary care physicians and specialists; reducing patient barriers to accessing primary care through insurance coverage, low deductibles, and convenience; and investing in health information technology to facilitate communication and access to patient information by all members of the care team.

There is strong evidence supporting interventions to improve providers’ ability to perform essential functions and reorienting health systems to encourage primary care; increasing numbers of primary care providers alone is less likely to achieve the best results.

Clalit Health Services’ accomplishments in achieving relatively high rates of continuity of care are likely attributable to a strong system orientation towards primary care and an adequate supply of trained primary care providers. System characteristics that reinforce primary care include delivery of most care through a network of primary care clinics, designation of a regular physician chosen by the patient, and zero copayments for primary care visits. Israel has placed emphasis on training adequate numbers of primary care providers as evidenced by the fact that the majority of patient visits are to primary care physicians, as opposed to specialists.

An important next step for Clalit Health Services will be to ascertain whether primary care providers are performing essential primary care functions and whether patients are achieving the best outcomes at lowest cost. In addition to utilization measures, the Dreiher study measured the association between continuity of care and use of a limited number of preventive services, and found small and mixed results. Additional analyses based on a more robust set of performance measures, many of which are available in Israel’s National Quality Measurement Program, will be needed to assess the level of quality of care and patient outcomes [9].

The “chronic care model” developed by Wagner et al. and adopted worldwide, provides an integrated framework to guide practice redesign [10]. The aim of the model is to improve patient outcomes by changing ambulatory care for patients with chronic illnesses from acute and reactive to proactive, planned, and population based. The model includes six components [11]:

· Self-management supports for patients and family caregivers.

· Decision-support to promote clinician and patient shared decision-making informed by the best evidence and patient preferences.

· Delivery system design including planned visits with follow-up, team-based care delivery, and care management programs for high risk patients.

· Health information systems including registry data to support proactive outreach to at-risk patients and planned visits (via e-mail and office), and performance measurement with real-time feedback.

· Health care organizations with a culture, mechanisms, and incentives aligned with patient-centered care and continuous improvement.

· Community resources, established through partnerships between medical and community-based organizations, to support patients and family caregivers in reducing health risks and implementing care plans.

A growing body of evidence substantiates the importance of each of these functions to achieving the best patient outcomes at the lowest cost.

Implementing all of the functions of the chronic care model is challenging, but Israel has already established a solid foundation and demonstrated important accomplishments. A strong commitment has been made to primary care as a central organizing concept of the health system, and important investments have been made in health information technology and training primary care physicians. Taking the next steps toward a comprehensive system of patient- and population-centered care for proactive management of chronic illnesses has great potential to enhance patient outcomes and reduce costs.

## Competing interest

Dr. Corrigan has no conflicts of interest to declare.

## **Author information**

Janet M. Corrigan, PhD, MBA, is president and CEO of the National Quality Forum (NQF), a not-for-profit standard-setting organization that endorses performance measures, and provides guidance to both the public and private sectors on use of measures in payment and public reporting programs. Dr. Corrigan received her doctorate in health services research and master of industrial engineering degrees from the University of Michigan, and master’s degrees in business administration and community health from the University of Rochester.
